# Functionalization of Human Serum Albumin by Tyrosine Click

**DOI:** 10.3390/ijms22168676

**Published:** 2021-08-12

**Authors:** Satsuki Obara, Keita Nakane, Chizu Fujimura, Shusuke Tomoshige, Minoru Ishikawa, Shinichi Sato

**Affiliations:** 1 Graduate School of Life Sciences, Tohoku University, 2-1-1 Katahira, Aoba-ku, Sendai 980-8577, Japan; satsuki.obara.s4@dc.tohoku.ac.jp (S.O.); nakane.keita.s1@dc.tohoku.ac.jp (K.N.); stomoshi@tohoku.ac.jp (S.T.); minoru.ishikawa.e4@tohoku.ac.jp (M.I.); 2 Frontier Research Institute for Interdisciplinary Sciences, Tohoku University, 2-1-1 Katahira, Aoba-ku, Sendai 980-8577, Japan; chizu.fujimura.c4@tohoku.ac.jp

**Keywords:** tyrosine click, human serum albumin, drug binding, laccase, modification site

## Abstract

Human serum albumin (HSA) is a promising drug delivery carrier. Although covalent modification of Cys34 is a well-established method, it is desirable to develop a novel covalent modification method that targets residues other than cysteine to introduce multiple functions into a single HSA molecule. We developed a tyrosine-selective modification of HSA. Three tyrosine selective modification methods, hemin-catalyzed, horseradish peroxidase (HRP)-catalyzed, and laccase-catalyzed reactions were performed, and the modification efficiencies and modification sites of the modified HSAs obtained by these methods were evaluated and compared. We found that the laccase-catalyzed method could efficiently modify the tyrosine residue of HSA under mild reaction conditions without inducing oxidative side reactions. An average of 2.2 molecules of functional groups could be introduced to a single molecule of HSA by the laccase method. Binding site analysis using mass spectrometry suggested Y84, Y138, and Y401 as the main modification sites. Furthermore, we evaluated binding to ibuprofen and found that, unlike the conventional lysine residue modification, the inhibition of drug binding was minimal. These results suggest that tyrosine-residue selective chemical modification is a promising method for covalent drug attachment to HSA.

## 1. Introduction

Serum albumin is the most abundant monomeric multidomain protein in plasma and contributes to the regulation of oncotic pressure and fluid distribution. Human serum albumin (HSA) is found in human serum at 35–50 g/L and is composed of 585 amino acid residues. HSA acts as a carrier for many endogenous and exogenous compounds, including fatty acids, bilirubin, uremic substances, nitrogen monoxide, calcium ions, hormones, and drugs. [1] This protein has excellent characteristics as a drug carrier as it has a long half-life and specific accumulation in tumor tissues [2,3,4,5]. Additionally, HSA has several binding pockets where various ligands can bind non-covalently. Therefore, several albumin-based therapeutics have been approved as clinical drugs. For example, Abraxane^®^, a nanoparticle formulation of paclitaxel and albumin, was first approved by the FDA in 2005 as a clinical treatment for metastatic breast cancer and has subsequently been used for treatment of small cell lung cancer and advanced pancreatic cancer [6,7]. Strategies other than non-covalent attachment of drugs to albumin include fusion proteins and covalent attachment, which have also been approved for clinical use [8,9]. Amino acid residue modification of HSA by covalent bonds provides many options for drug targeting. The most commonly used method is the modification of Cys34, which is the only free cysteine in HSA. Cys34 is located on the protein surface and provides a unique thiol group for site-specific covalent binding of the drug of interest. To attach multiple drugs into one HSA molecule, the development of methods other than Cys34 modification is required.

HSA has 59 lysine residues and can be used as a scaffold for the covalent modification of drugs. In addition, some compounds with electrophilic properties, such as β-lactam antibiotics, have been reported to react with lysine residues present on the surface of HSA [10]. Lysine-modified albumins with an electrically charged boron cluster have been developed for boron neutron capture therapy, in these studies modified albumins were used as a carrier of boron to tumor tissue [11,12,13,14]. Barbas et al. developed a method to selectively modify Lys64 using a ligand-directed strategy [15]; as lysine residues are charged by NH_3_^+^ groups, acylation can neutralize this charge to conformationally alter the modification site [16]. Modification of lysine residues inhibits drug binding, which may impair the original function of HSA [17,18]. This property can be a disadvantage of the lysine modification method.

On the other hand, we have developed methods to modify tyrosine residues using hemin, HRP, and ruthenium photocatalysts [19,20,21,22,23]. More recently, we have shown that laccase can efficiently modify tyrosine residues under mild conditions using dissolved oxygen as the driving force for oxidation [24]. Although attempts have been made to develop methods for modifying tyrosine residues in HSA [17], it has been difficult to efficiently functionalize tyrosine residue selectively. In this study, we applied our original tyrosine residue modification to HSA modification. To multi-functionalize HSA, tyrosine residue-specific modification can introduce functional groups into HSA without damaging the cysteine or lysine residues, and thus can be used orthogonally to conventional methods.

## 2. Results

### 2.1. Selection of Conditions for Tyrosine Residue Modification of HSA

**Figure 1:**
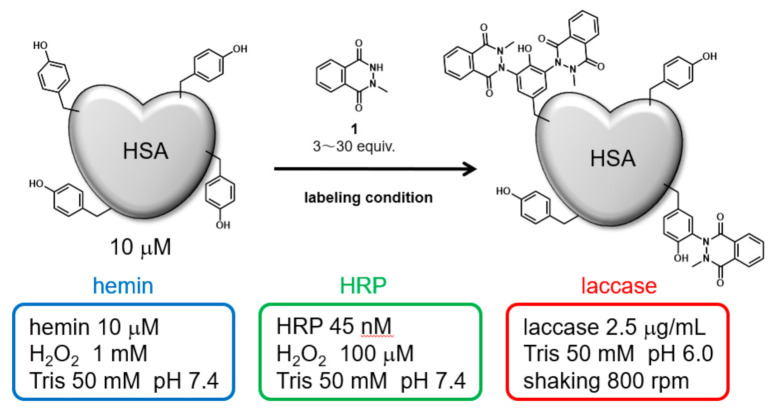
HSA modification by *N*-methylated luminol derivative (**1**) and three catalytic reaction conditions.

Initially, HSA was modified under three catalytic reaction conditions: hemin, HRP, and laccase. The modification reagent, *N*-methylated luminol derivative (**1**), was added at concentrations from 3 to 30 equivalents for 10 μM HSA (Figure 1). The modified HSAs were fragmented into peptides by sodium dodecyl sulfate polyacrylamide gel (SDS-PAGE) followed by gel trypsin digestion, and the modified amino acid residues were detected by nanoscale liquid chromatography coupled to tandem mass spectrometry (nanoLC-MS/MS). We conducted a search for modified peptide on tyrosine, cysteine, tryptophan, and histidine residues, suggesting that only tyrosine residues were modified. A quantitative comparison of the modification efficiency of each labeling site among the modification conditions was also performed based on the peptide peaks detected by the Q-TOF system (See Supporting Information Figures S1–S14 for MS/MS analysis attribution).

### 2.2. Identification of Modification Sites by Mass Spectrometry and Comparison of Modification Efficiency under Various Reaction Conditions

**Figure 2:**
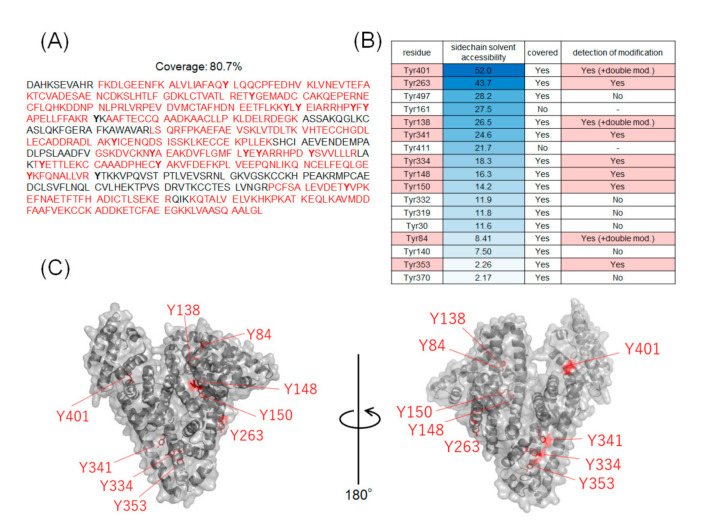
Tyrosine residues in modified HSA. (**A**) Amino acid sequences of HSA and peptide fragments detected by nanoLC-MS/MS analysis. (**B**) Sidechain solvent accessibility of tyrosine residues and labeling sites. The peptide fragment containing Tyr161 and Tyr411 were not detected. (**C**) Mapping of modified tyrosine residues on 3D structures (PDB:1BM0).

**Figure 3:**
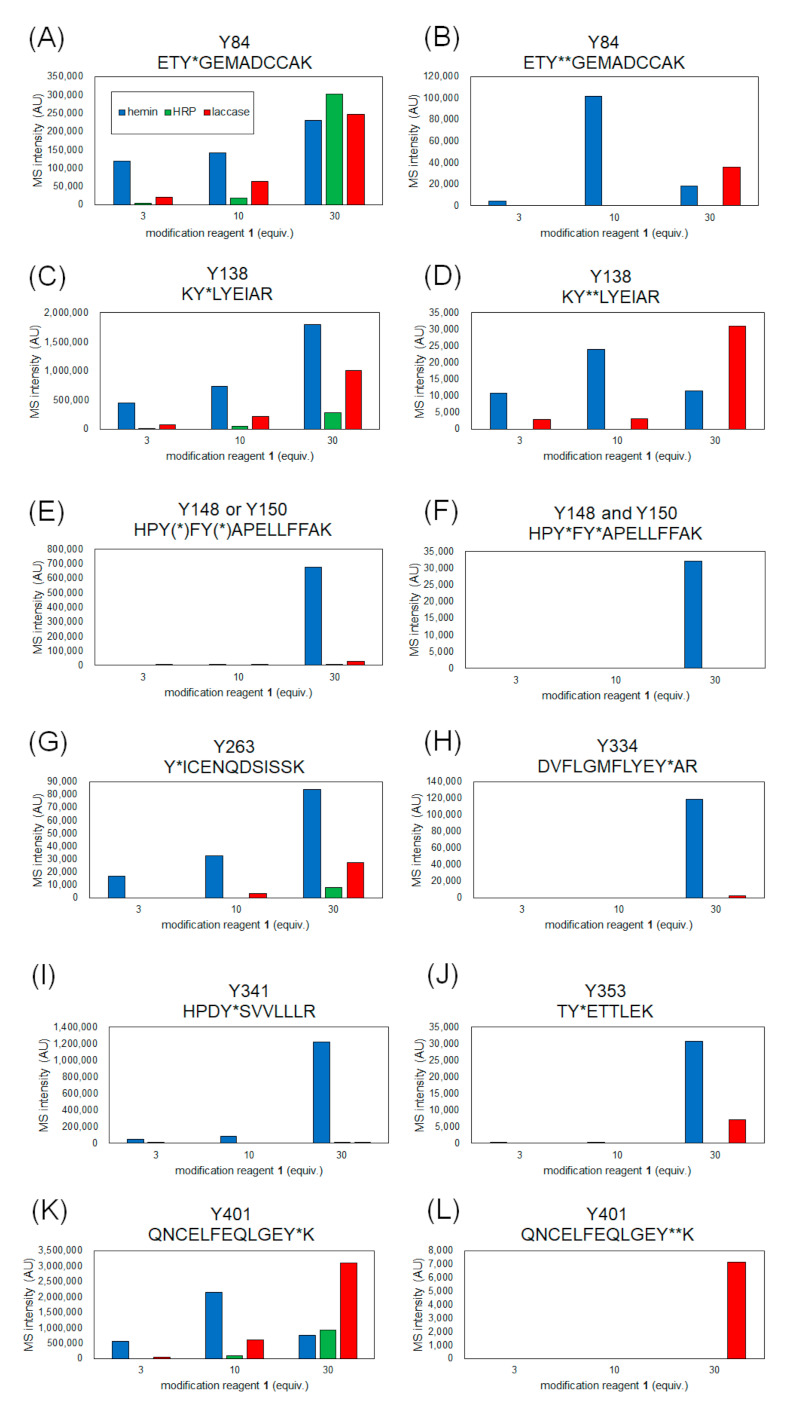
MS intensity comparison of modified peptides containing each tyrosine residue. Y* and Y** indicate single and double modified tyrosines with **1**. Peptide fragment containing modified Tyr84 (**A**), Tyr138 (**C**), Tyr148 or/and Tyr150 (**E**,**F**), Tyr263 (**G**), Tyr334 (**H**), Tyr341 (**I**), Tyr353 (**J**), Tyr401 (**K**) and double-modified Tyr 84 (**B**), Tyr138 (**D**), Tyr401 (**K**,**L**).

**Figure 4:**
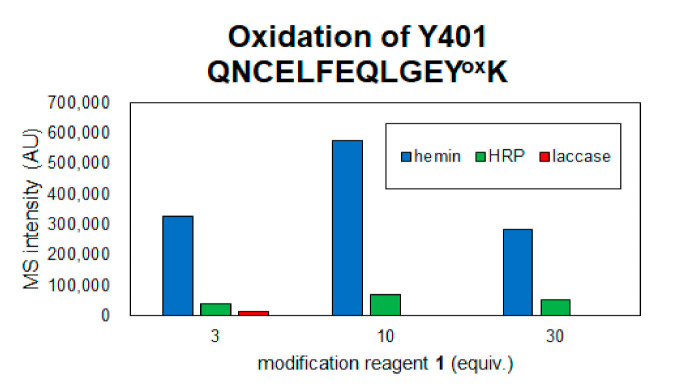
Detection of oxidized peptide of Y401 in each reaction condition.

The coverage of the peptide fragments detected by nanoLC-MS/MS analysis is shown in Figure 2A,B. Focusing on the tyrosine residues, peptide fragments containing 15 tyrosine residues other than Tyr161 and Tyr411 were detected among the 17 tyrosine residues in HSA. Tyrosine residues modified under any of the catalytic reaction conditions are highlighted in Figure 2B,C. We have also reported that under HRP conditions, tyrosine modification tends to occur preferentially on tyrosine residues exposed on the protein surface. Therefore, we sorted the tyrosine residues in Figure 2B based on their sidechain solvent accessibility. Sidechain solvent accessibility indicates the surface area of amino acid sidechain that is accessible to a solvent, and is a parameter that determines their surface exposure level. Figure 2B shows that each tyrosine residue is calculated from the protein structure obtained X-ray crystal structure of HSA (PDB: 1BM0). The modification efficiency of each modified tyrosine residue is shown in Figure 3. Because the intensity of ionization is different for peptide fragments with different sequences, it is not possible to make a quantitative site-by-site comparison from the detected peak intensities, but it is possible to compare the modification efficiency for each site for each condition. Figure 4 shows a comparison of the peak intensities of the oxidized form of Y401 as an example of an oxidative side reaction. Differences in the efficiency of modification and oxidation for different conditions were discussed in detail in the Section 3.

### 2.3. Modification with Azide-Conjugated Modification Reagent and Evaluation of Modification Efficiencies

**Figure 5:**
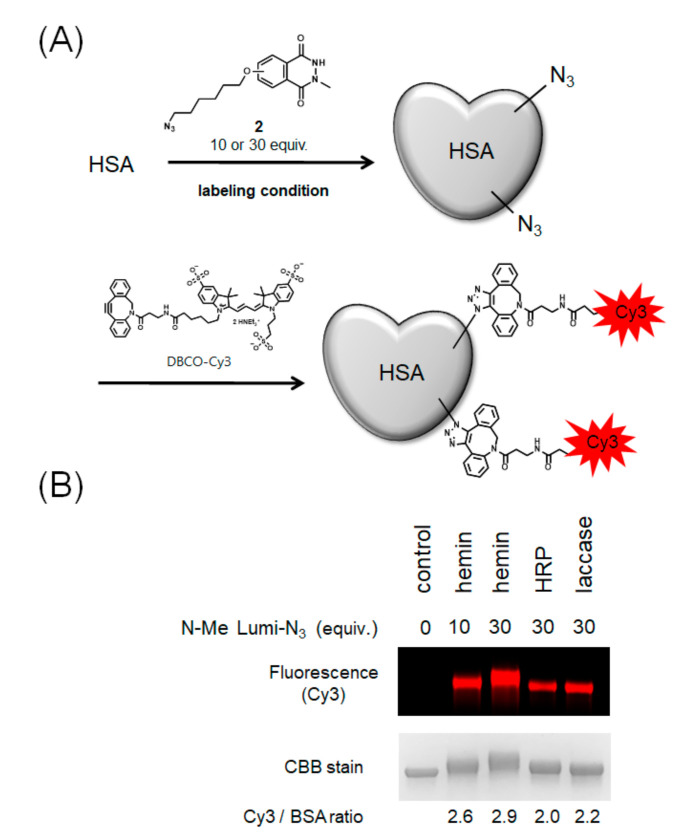
Modification of HSA with azide-conjugated *N*-methylated luminol derivative (**2**) and comparison of modification efficiencies. (**A**) Scheme of HSA modification using **2** and DBCO-Cy3. (**B**) Comparison of modification efficiency from Cy3 fluorescence intensity. N = 2. Representative fluorescent gel is shown in Figure 5B.

Next, we performed HSA modification with azide-conjugated *N*-methylated luminol derivative **2**, which can introduce an arbitrary functional group by the click reaction (Figure 5A). To visualize the efficiency of azide group modification, we modified HSA with Cy3 by a Cu-free click reaction using dibenzocyclooctyne (DBCO) conjugated-Cy3 and calculated the number of Cy3 molecules bound to one molecule of HSA by fluorescence detection of the resulting modified protein. The results showed that hemin gave the most efficient modification followed by laccase with high efficiency (Figure 5B).

### 2.4. Evaluation of Drug Binding Ability of Tyrosine-Modified HSA

**Figure 6:**
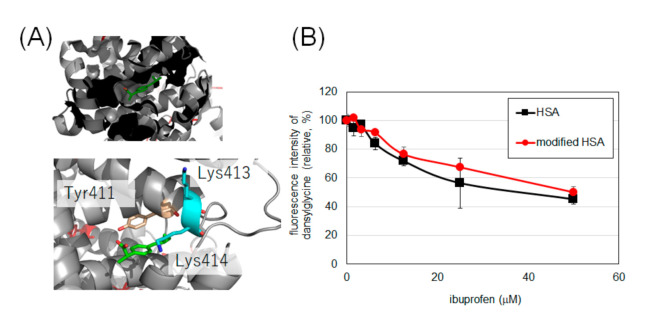
Binding of HSA and modified HSA to ibuprofen. (**A**) The binding of ibuprofen at Sudlow’s site II (PDB: 6U4X). (**B**) Fluorescence changes due to competition between ibuprofen and dansylglycine. Error bars, mean ± SD from three independent experiments. These experiments were performed at 25 °C (See Figure S16 for temperature-dependency).

Considering the low oxidative damage to HSA and the reasonable modification efficiency, we set the modification condition using laccase and 30 equivalents of **2** as the optimal conditions to modify HSA and evaluate the drug-binding ability of tyrosine-modified HSA. It has been reported that the binding of ibuprofen and HSA to Sudlow’s site II can be measured by the fluorescence intensity of dansylglycine (See Supporting Information Figure S15 for the change in fluorescence spectrum of dansylglycine by binding with HSA) [25,26]. Dansylglycine antagonizes ibuprofen at the binding site, resulting in reduced fluorescence. The fluorescence attenuations of dansylglycine to ibuprofen in HSA and modified HSA were comparable, suggesting that the drug-binding property of Sudlow’s site II was retained after modification with **2** (Figure 6B).

## 3. Discussion

From the results of mass spectrometry in Figure 2 and Figure 3, we discuss the differences in the modification sites and modification efficiency. The efficiency of the modification of laccase and HRP was found to increase in a concentration-dependent manner, whereas the detection of modified peptides for hemin decreased after treatment with a high concentration of **1** (Figure 3B,D,K). These phenomena might be resulting from competition between the tyrosyl radical-mediated modification and single electron oxidation of **1**. When hemin is used as a catalyst, the production of tyrosyl radicals is considered to be involved in the modification reaction. The addition of an excessive amount of **1** may competitively inhibit the tyrosyl radical generation. When the oxidation reaction of Y401 was monitored as a side reaction of the modification reaction, higher oxidation peaks were detected under the conditions using 10 equivalents of **1** than 30 equivalents of **1** (Figure 4).

Tyr138 is located at the hemin-binding site of HSA, and Tyr84 is present in close proximity to the binding site. Compared to the other catalysts, the modification reaction of hemin was observed at low concentrations, which may be due to the efficient modification of these tyrosine residues by the close proximity of the catalyst and the reaction site (Figure 3A–D). In these residues, a structure with two **1** molecules attached to one tyrosine residue was detected (double modification; Figure 3B,D). The phenolic hydroxyl group of Tyr84 forms a hydrogen bond with the SH group of Cys34 [27], which causes the residue to face the inside of the protein, resulting in low solvent accessibility (Figure 2B). However, under single-electron oxidation conditions, Cys34 can be radicalized, so its hydrogen bond is resolved, leading to higher exposure of Tyr84 than the X-ray structure under the reaction conditions. Additionally, electron transfer via the Cys34 radical may also accelerate the modification of Tyr84 (Figure 3A,B).

We found that relatively low exposure of amino acid residues, such as Tyr148, Tyr150, Tyr334, Tyr341, and Tyr353, caused the reaction by HRP and laccase to be less efficient than that of hemin (Figure 3E,F,H–J). This may be due to the fact that hemin is a hydrophobic structure that binds to the protein surface and catalyzes the reaction in close proximity to the tyrosine residue at the reaction site, whereas the enzymes HRP and laccase are less accessible to the partially exposed tyrosine residue, resulting in lower reaction efficiency. For Tyr263 and Tyr401, which have high solvent accessibility, modification was confirmed under reaction conditions using HRP and laccase in addition to hemin (Figure 3G,K,L). double modifications were detected in Tyr401, especially under laccase conditions (Figure 3L).

Considering the oxidation of amino acid residues as a side reaction, hemin seems to induce the oxidation of amino acid residues at a higher rate than the other two methods, as described above in Figure 4, where the oxidation of Tyr401 was detected, the oxidation level was hemin > HRP > laccase. In the laccase condition, oxidation was detected at three equivalents of **1**, but at 10 and 30 equivalents of **1**, the oxidation peak of Tyr401 was not detected because the single-electron oxidation of **1** competed with the oxidation of tyrosine residue and the trapping reaction of tyrosyl radical by **1** may contribute to the decrease in the side reaction. Mechanistically, unlike hemin and HRP, which require the addition of H_2_O_2_, laccase requires mild reaction conditions and is driven by O_2_ in the buffer. Therefore, the reaction conditions using laccase and 30 equivalents of **2** were determined as the optimal tyrosine residue modification conditions for the functional modification of HSA.

We elucidated whether the functionality of HSA was damaged by laccase modification using indicators other than residue oxidation. It has been reported that lysine residue modification causes a decrease in α-helix content and a decrease in binding of indomethacin [28] to HSA at Sudlow’s site I [29]. Binding of caprofen, which has been reported to bind to Sudlow’s site I and site II [30], is also reported to be reduced by lysine modification of HSA [17]. Ibuprofen has also been reported to bind to Sudlow’s site II [30], with Tyr411, Lys413, and Lys414 in the vicinity of the site (Figure 6A). It has been reported that radical species induced by metal-catalyzed oxidation attenuate the binding of ibuprofen to site II [31], there is concern that excessive oxidation conditions may reduce the binding capacity of ibuprofen. Therefore, we evaluated the binding of intact HSA and HSA modified with **2** to ibuprofen under laccase conditions, a mild single-electron transfer condition.

The results in Figure 6 confirm that HSA retains its binding to ibuprofen even after tyrosine modification. Although Tyr411 at this site is an uncovered residue in MS, we believe that the modification of this site did not affect the binding of ibuprofen, or that the binding of ibuprofen was not impaired because Tyr411 was not modified by this condition.

## 4. Material and Methods

### 4.1. HSA Modification Using Hemin

A solution of the modification reagents [20] **1** or **2** (from 3–30 mM solution in DMSO, final concentration 30, 100, or 300 μM) was added to HSA solution (Sigma-Aldrich, St. Louis, USA, final concentration 10 μM in 50 mM Tris buffer [pH7.4]). Hemin (Sigma-Aldrich, final concentration 10 µM) and H_2_O_2_ (final concentration 1 mM) were added to the mixture, which was then incubated at room temperature for 1 h. Next, the excess modification reagents were removed by centrifuging at 2000× *g* for 4 min using a Sephadex G-25 gel (GE Healthcare, Chicago, USA) filtration column. 

### 4.2. HSA Modification Using HRP

A solution of the modification reagents **1** or **2** (from 3–30 mM solution in DMSO, final concentration 30, 100, or 300 μM) was added to HSA solution (Sigma-Aldrich, final concentration 10 μM in 50 mM Tris buffer [pH7.4]). HRP (Sigma-Aldrich, final concentration 45 nM) and H_2_O_2_ (final concentration 25 µM) were added to the mixture, which was then was incubated at room temperature for 1 h. Next, the excess modification reagents were removed by centrifuging at 2000× *g* for 4 min using a Sephadex G-25 gel (GE Healthcare) filtration column [21]. 

### 4.3. HSA Modification Using Laccase

A solution of the modification reagents **1** or **2** (from 3–30 mM solution in DMSO, final concentration 30, 100, or 300 μM) was added to HSA solution (Sigma-Aldrich, final concentration 10 μM in 50 mM Tris buffer [pH 6.0]). Laccase (Amano Enzyme Inc., Aichi, Japan, final concentration 2.5 μg/mL) was added to the mixture and the mixture was shaken at 800 rpm (using a Thermo shaker) at 37 °C for 1 h. After 1 h, excess modification reagents were removed by centrifuging at 2000× *g* for 4 min using a Sephadex G-25 gel (GE Healthcare) filtration column [24]. 

### 4.4. In Gel Digestion

HSAs modified with **1** were obtained using the protocol shown in Section 4.1, Section 4.2 and Section 4.3. The modified HSA solution was added to 5× sample buffer (final concentration 50 mM; Tris-HCl [pH 6.8], 2% sodium dodecyl sulfate [SDS], 0.025% bromophenol blue, and 10% glycerol), heated at 95 °C for 5 min, and separated by SDS-PAGE using 4–20% acrylamide gels (Bio-Rad). Bands corresponding to modified HSAs were separated and excised (ca. 1 mm pieces). Gel pieces were transferred into microtubes, and 1 mL of water was added to the tubes and incubated at 37 °C for 10 min. The solution was removed, and the washing procedure was repeated thrice. For de-staining, 50% CH_3_CN in aqueous NH_4_HCO_3_ solution (100 mM) was added and incubated at 37 °C for 10 min, and the solution was removed. Next, CH_3_CN was added to the tubes for dehydration and incubated at 37 °C for 10 min, and the solution was removed. To the tubes, 100 mM dithiothreitol in aqueous NH_4_HCO_3_ solution (100 mM) was added for Cys reduction, incubated at 37 °C for 30 min, and the solution was removed. Next, 250 mM iodoacetamide in aqueous NH_4_HCO_3_ solution (100 mM) was added to the tubes for Cys alkylation, followed by incubation at room temperature for 30 min in the dark, after which the solution was removed. Then, an aqueous NH_4_HCO_3_ solution (100 mM) was added to the tubes and removed, followed by the addition of 50% CH_3_CN in aqueous NH_4_HCO_3_ solution (100 mM). After the solution was removed, trypsin solution was added to each tube and incubated overnight at 37 °C. The resulting solution was quenched with aqueous TFA solution (final concentration 0.1% *v*/*v*) and desalted using C18 pipette tips (Nikkyo Technos Co., Ltd. Tokyo, Japan). The desalted solution was subjected to nanoLC-MS/MS analysis.

### 4.5. NanoLC-MS/MS Analysis 

NanoLC-MS/MS analysis was performed by LC-nano-ESI-MS comprising a quadrupole time-of-flight mass spectrometer (Triple TOF^®^ 5600 system; SCIEX) equipped with a nanospray ion source and a nanoLC system (Eksigent Nano LC Ultra 1D Plus; SCIEX, Massachusetts, U.S.A.). The trap column used for the nanoLC was a NanoLC Trap ChromXP C18, 3 μm 120 Å (SCIEX) and the separation column was a 12.5 cm × 75 μm capillary column packed with 3 μm C18-silica particles (Nikkyo Technos Co., Ltd., Japan). The micropump (flow rate 300 nL/min) gradient method was used as follows: Mobile phase A: 2% acetonitrile, 0.1% formic acid, mobile phase B: 80% acetonitrile, 0.1% formic acid aq. 0–20 min: 5–45% B, 20−21 min: 45–100% B, 21–26 min: 100% B. The nanoLC-MS/MS data were acquired in information-dependent acquisition mode controlled by Analyst^®^ TF 1.5.1 software (SCIEX). The settings of data-dependent acquisition were as follows: accumulation time was 0.25 s; the full MS (MS1, TOF-MS) scan range was 400–1250 m/z; exclude former target ion for 12 s; mass tolerance 50 mDa; and the top 10 signals were selected for MS2 scan per one full MS scan. MS2 (Product ion) scan accumulation time and range were 0.05 s and 100–1500 m/z. The experiment was conducted in triplicate. The MS/MS spectra were searched against the respective amino acid sequence (HSA) using MaxQuant (freeware) [32] with a default setting. A FASTA file corresponding to the HSA sequence was used. For the modification settings, oxidation (+ O) for His, Met, Tyr, and Trp residues; deoxidation (+ O_2_) for His, Met, and Trp; oxidation with hydrogen loss (+O -H_2_) for Tyr and His; carbamidomethyl (+CHNO) for Cys residue; acetylation (+C_2_H_2_O) for N-terminal adduct of 1 (+C_9_H_6_N_2_O_2_; + 174.043 Da) for His, Trp, Cys, and Tyr residues; and double adduct of 1 (+C_18_H_12_N_4_O_4_; + 348.086 Da) for Tyr residues were set as variable modifications. 

### 4.6. Evaluation of Labeling Efficiency

DBCO-Cy3 (from 10 mM solution in DMF, final concentration 100 µM) was added to a solution of azide-labeled HSA and incubated for 30 min at 37 °C. After 30 min, excess modification reagents were removed using a Sephadex G-25 gel (GE Healthcare) filtration column (2000× *g*, 4 min). The amount of Cy3 modified on the protein was calculated by measuring the fluorescence intensity of the Cy3 modified protein using a plate reader (Infinite F PLEX, TECAN, Männedorf, Switzerland). The modified HSA solution was added to 5× SDS-PAGE sample buffer, and the sample was heated at 95 °C for 5 min. The protein concentration was determined by the band intensity of the CBB-stained SDS-PAGE gels (4–20% acrylamide gels, Bio-Rad) compared with that of HSA samples of known concentration and quantified by ImageJ. The amount of Cy3 modification per protein molecule of the reference Cy3-modified protein was determined and used as a standard. The amount of Cy3 modification per molecule of HSA was determined by measuring the fluorescence intensity ratio against the standard sample on SDS-PAGE gels using a gel imager Fusion Solo S (VILBERLOURMAT, Collégien, France). 

### 4.7. Evaluation of Ligand Binding Ability of Labeled HSA

HSA (100 mM PBS [pH7.4], final concentration 5 μM) or modified HSA (prepared as in Section 4.3 and diluted in 100 mM PBS [pH7.4], final concentration 5 μM) was mixed with dansylglycine (from 100 mM solution in DMSO, final concentration 80 μM) and ibuprofen (from 0.156–5.0 mM solution in DMSO, final concentration 1.56−50 μM) in 100 mM PBS buffer (pH 7.4, final 1% DMSO). After 5 min of incubation at room temperature, the fluorescence intensity of dansylglycine (Ex: 360 ± 35 nm, Em: 465 ± 35 nm) was measured using a plate reader (Infinite F PLEX, TECAN). Error bars, mean ± SD from three independent experiments.

## 5. Conclusion

Modification of HSA with tyrosine residues can be an alternative covalent modification method for Cys34 modification. In this study, we compared three methods of modification: hemin-catalyzed, HRP-catalyzed, and laccase-catalyzed reactions, considering the high modification efficiency and tyrosine residue selectivity. It was found that the laccase-catalyzed method could efficiently modify the tyrosine residue of HSA under mild reaction conditions without inducing oxidative side reactions. An average of 2.2 molecules of functional groups could be introduced to a single molecule of HSA by the laccase method. Binding site analysis using mass spectrometry suggested Y84, Y138, and Y401 as the main modification sites. Furthermore, unlike the conventional lysine residue modification, inhibition of ibuprofen binding was minimal. These results suggest that tyrosine-residue selective chemical modification is a promising method for covalent drug attachment to HSA.

## Data Availability

Not applicable.

## Supplementary Materials

The following are available online at https://www.mdpi.com/article/10.3390/ijms22168676/s1.
