# Supplementary material for: Functionalization of Human Serum Albumin by Tyrosine Click
Source: Int J Mol Sci. 2021 Aug 12;22(16):8676. doi: 10.3390/ijms22168676 (PMC8395410; doi:10.3390/ijms22168676)
Supplement: Supplementary file 1 [file ijms-22-08676-s001.zip › ijms-1313708-supplementary.pdf]

Supporting Information

Functionalization of Human Serum Albumin by Tyrosine Click

Satsuki Obara,[a‡] Keita Nakane,[a‡] Chizu Fujimura,[a] Shusuke Tomoshige,[a] Minoru Ishikawa,[a] Shinichi Sato*[a,b]

a) Graduate School of Life Sciences, Tohoku University

2-1-1 Katahira, Aoba-ku, Sendai 980-8577, Japan

b) Frontier Research Institute for Interdisciplinary Sciences, Tohoku University

‡These authors contributed equally.

List of contents

1. Supporting Data S2–S17

1. Supporting Data

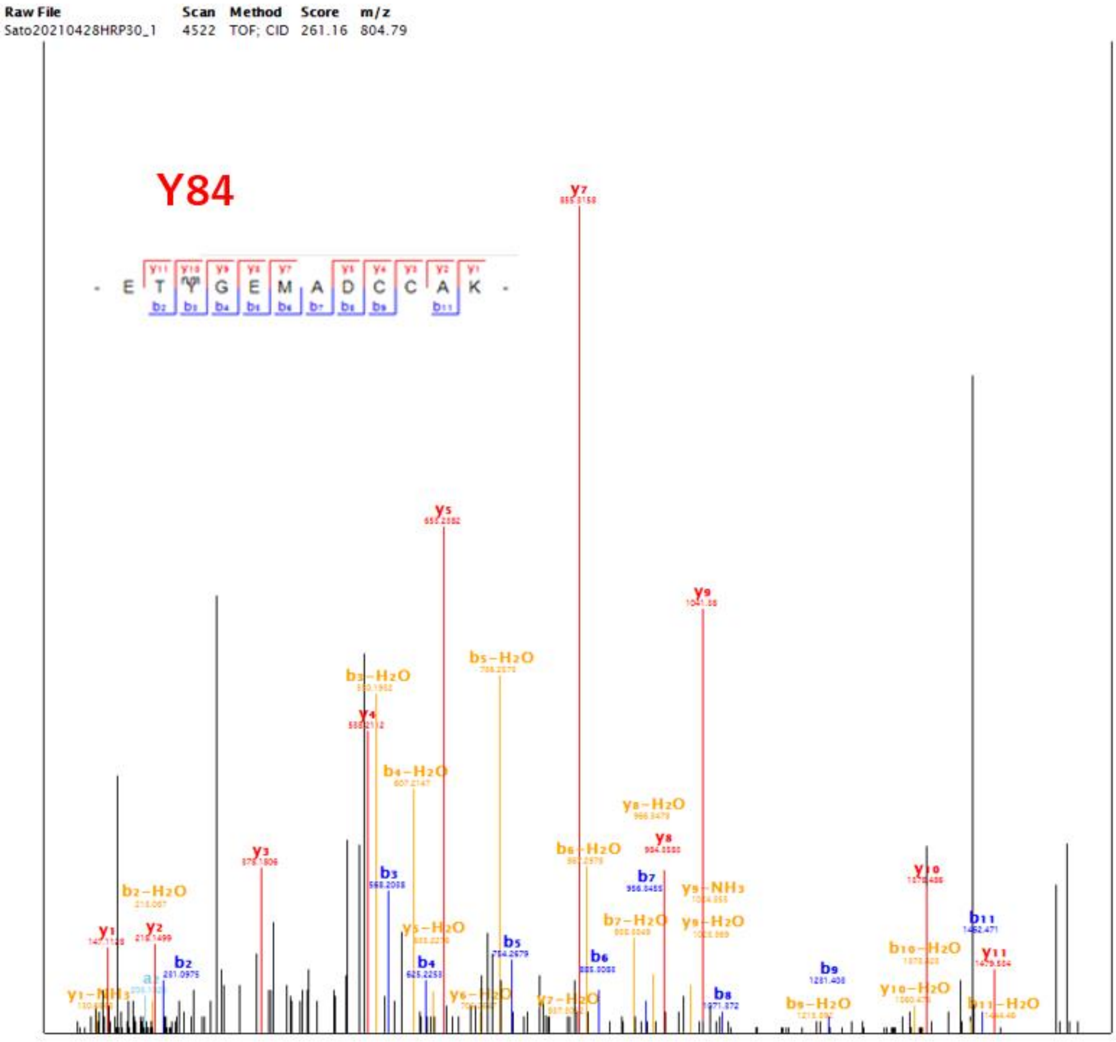

Figures S1. MS/MS analysis of the peak containing modified tyrosine shown in Figure 3A.

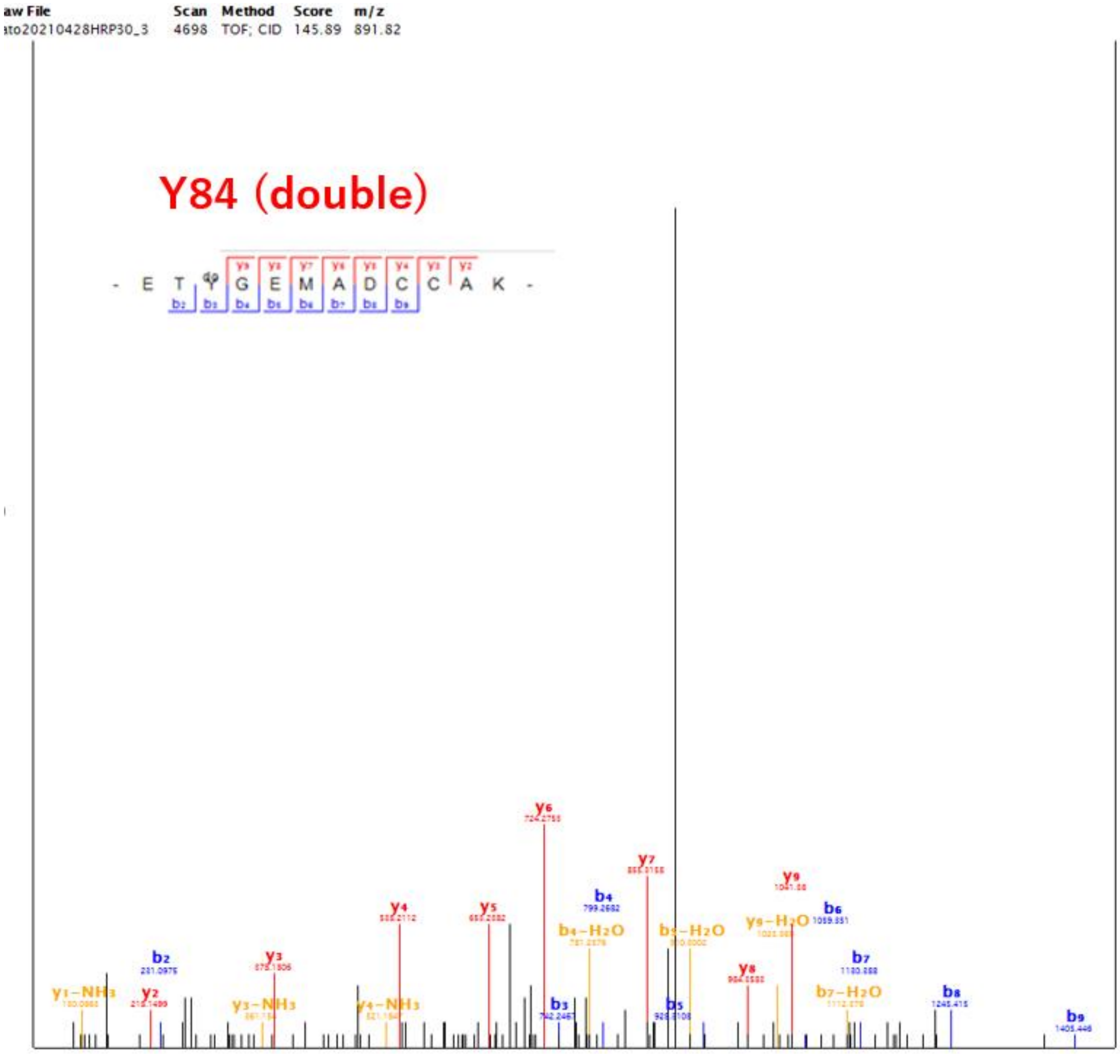

Figures S2. MS/MS analysis of the peak containing modified tyrosine shown in Figure 3B.

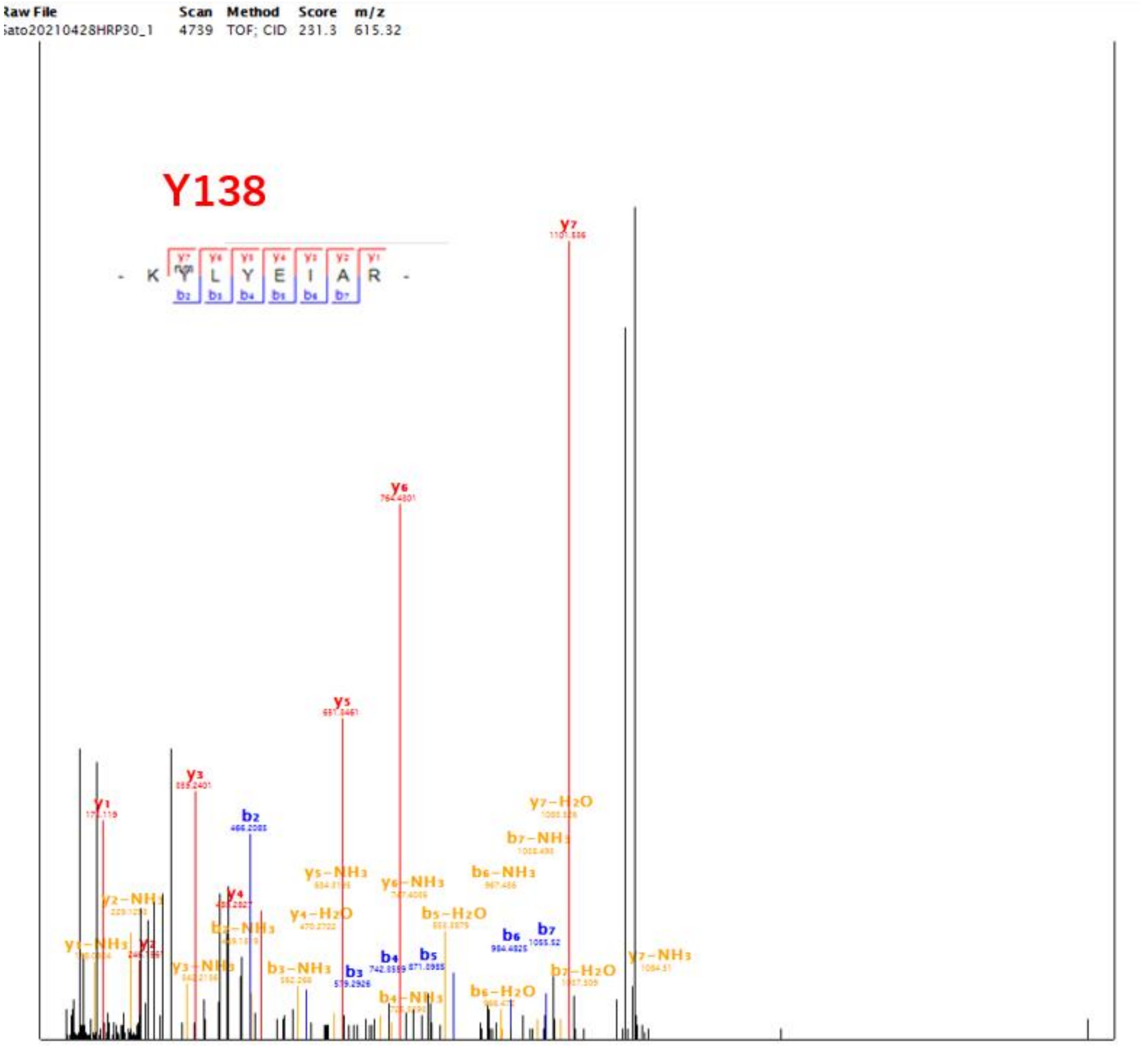

Figures S3. MS/MS analysis of the peak containing modified tyrosine shown in Figure 3C.

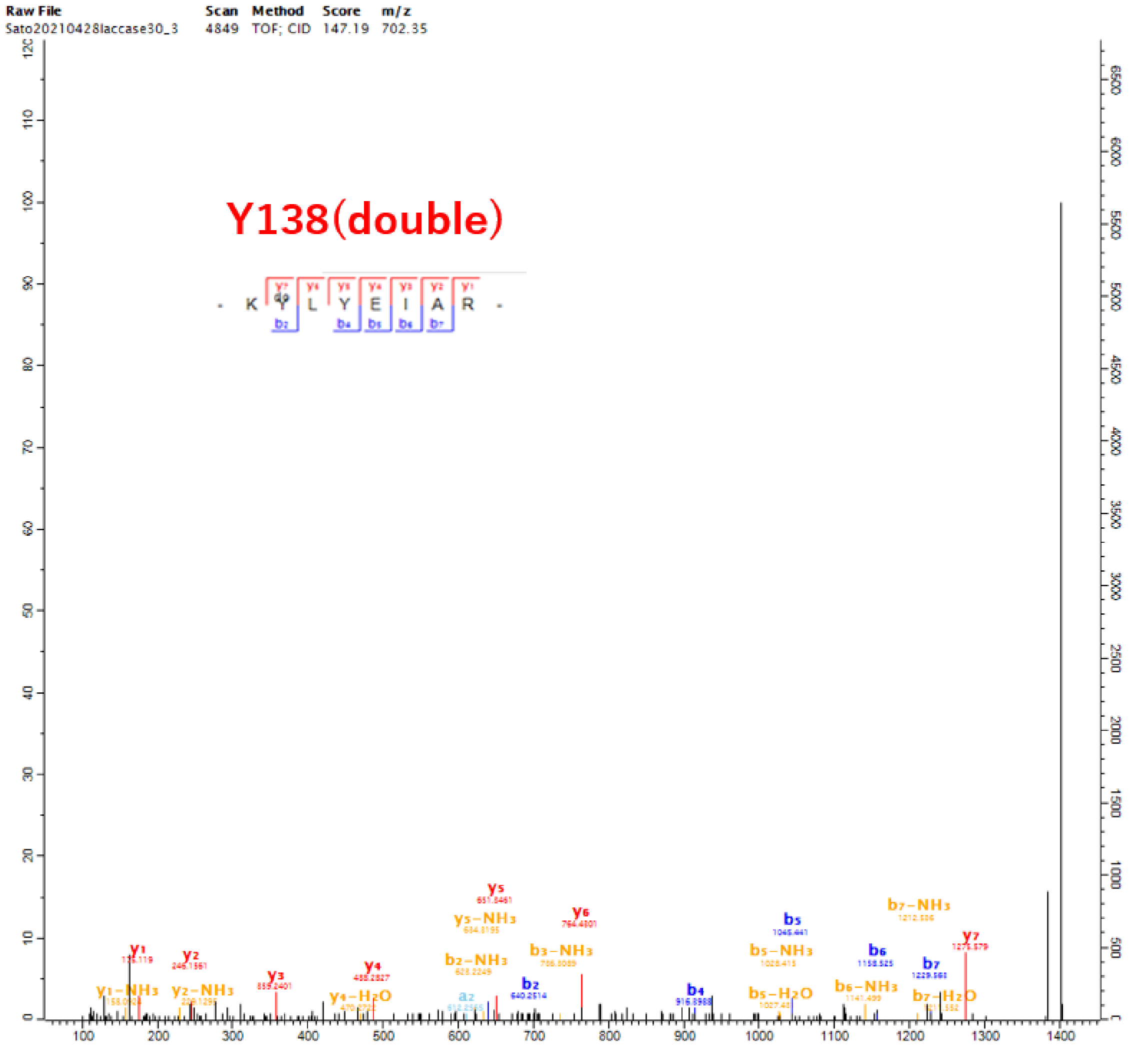

Figures S4. MS/MS analysis of the peak containing modified tyrosine shown in Figure 3D.

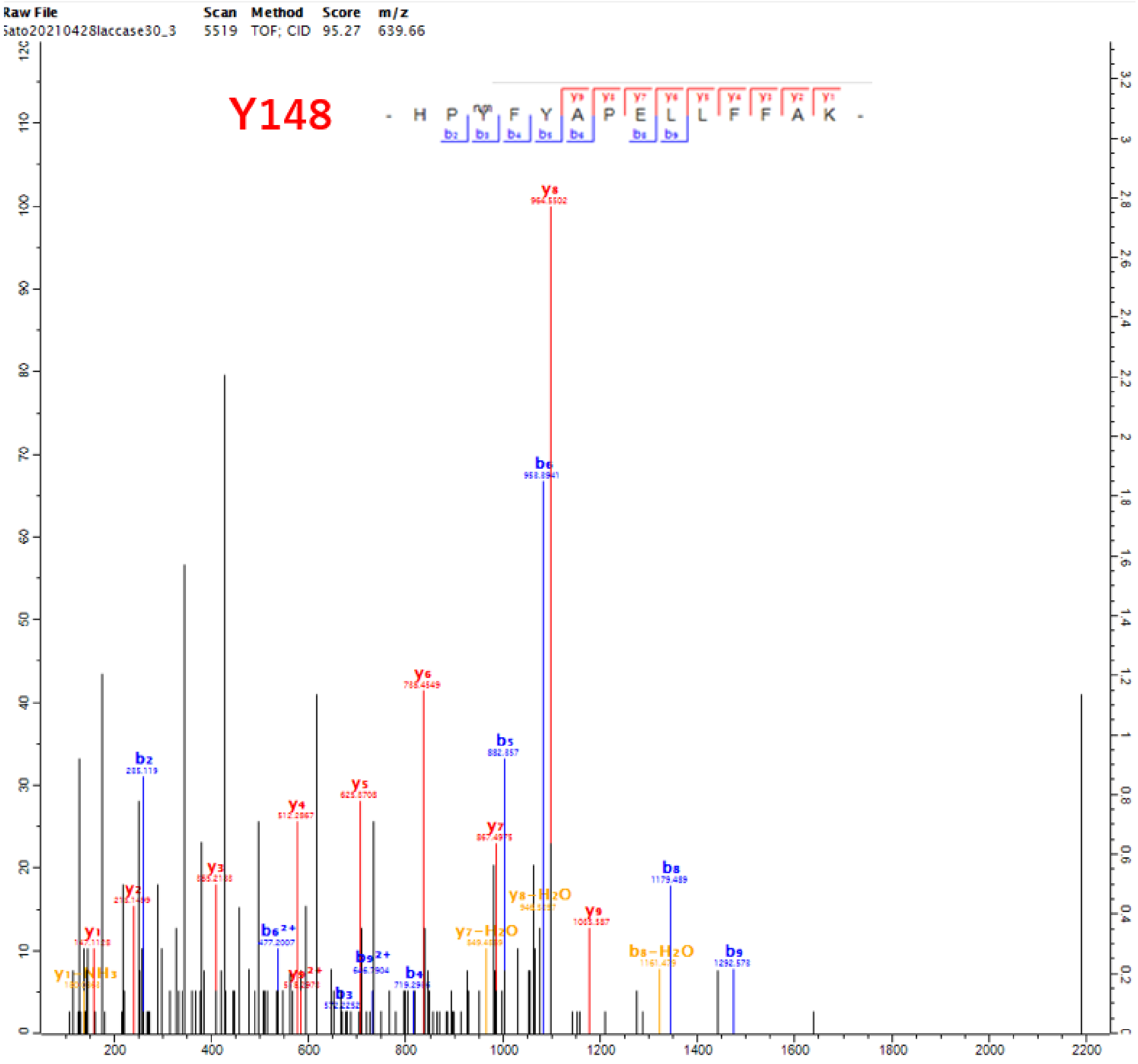

Figures S5. MS/MS analysis of the peak containing modified tyrosine shown in Figure 3E.

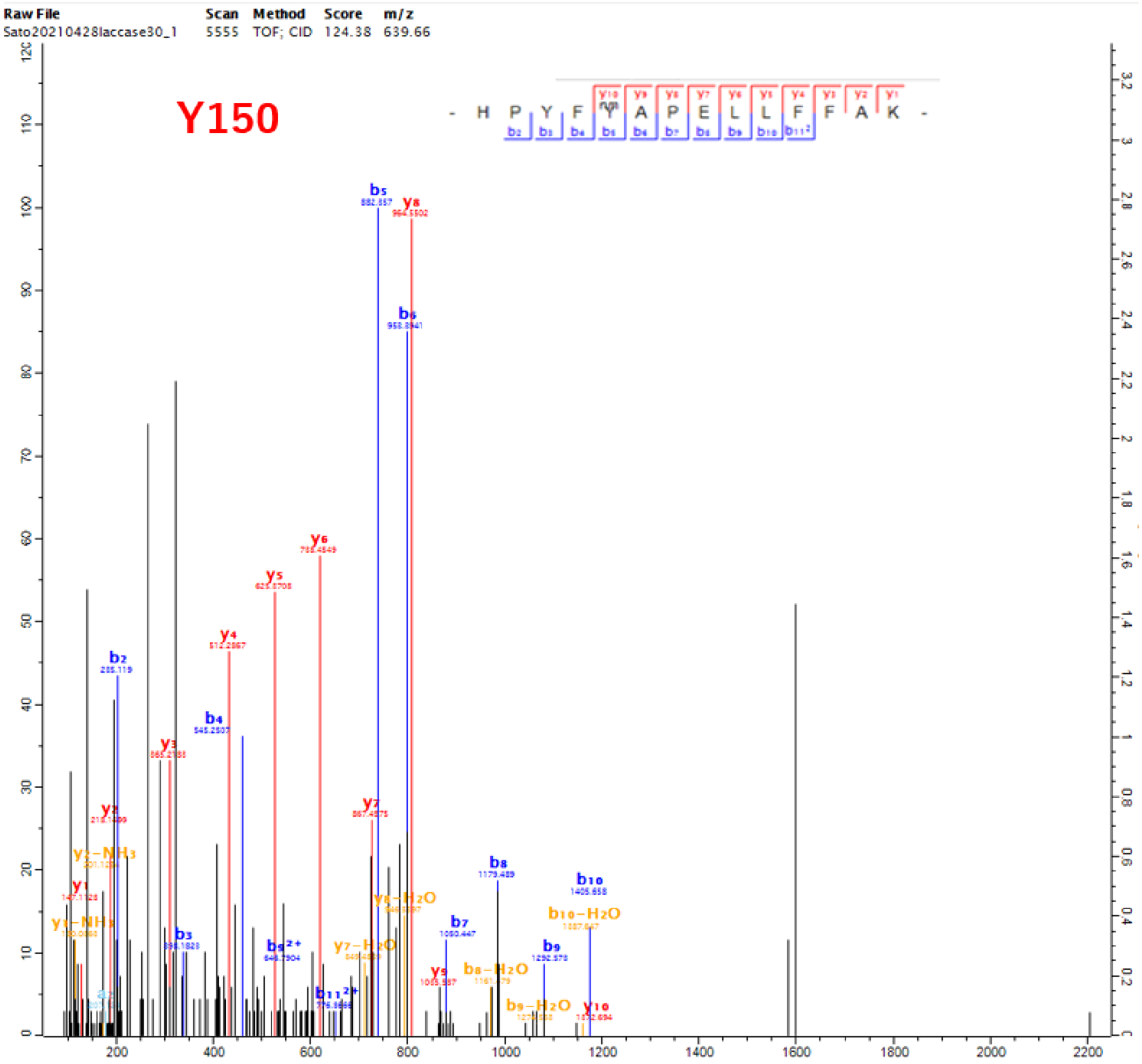

Figures S6. MS/MS analysis of the peak containing modified tyrosine shown in Figure 3E.

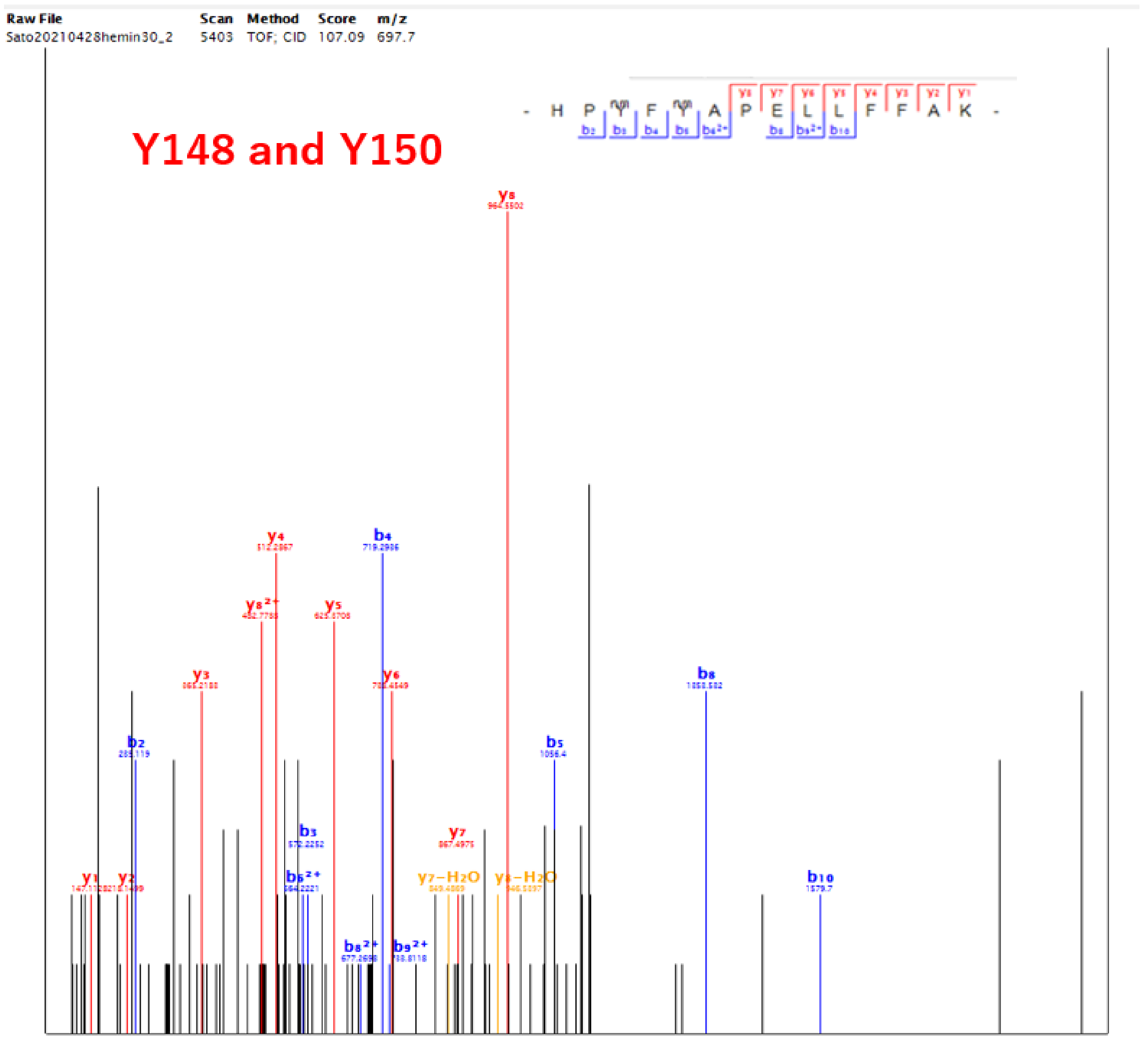

Figures S7. MS/MS analysis of the peak containing modified tyrosine shown in Figure 3F.

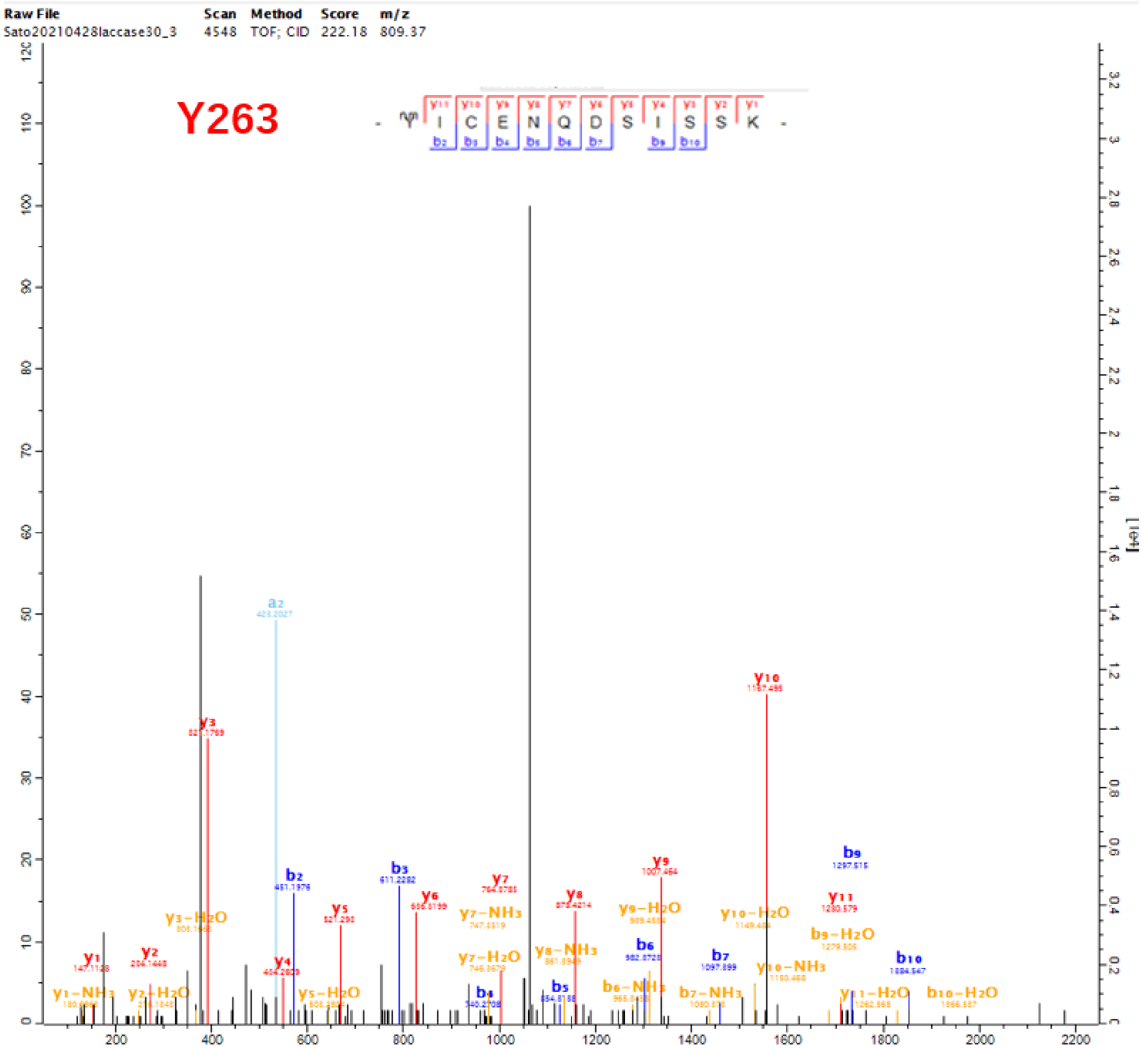

Figures S8. MS/MS analysis of the peak containing modified tyrosine shown in Figure 3G.

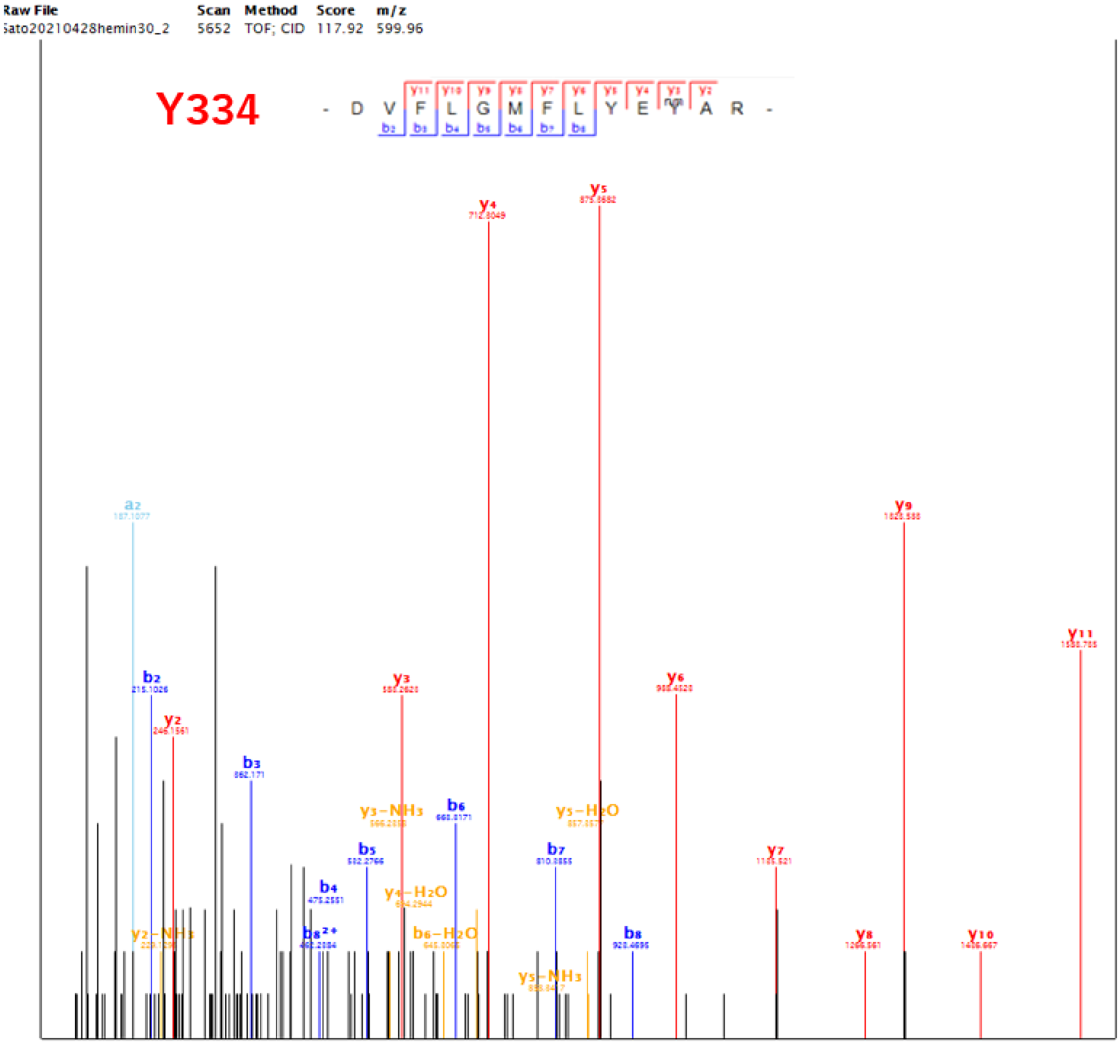

Figures S9. MS/MS analysis of the peak containing modified tyrosine shown in Figure 3H.

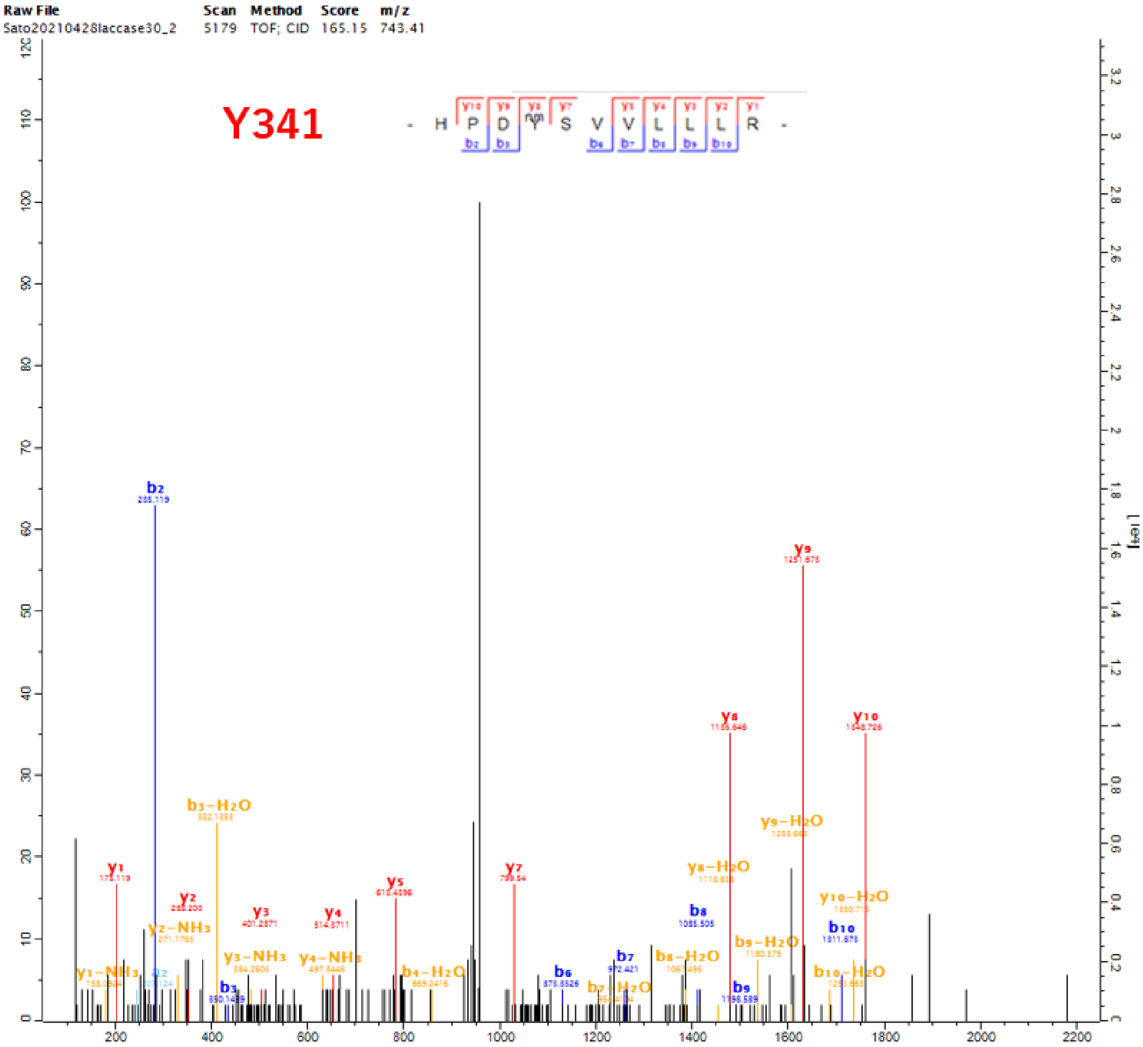

Figures S10. MS/MS analysis of the peak containing modified tyrosine shown in Figure 3I.

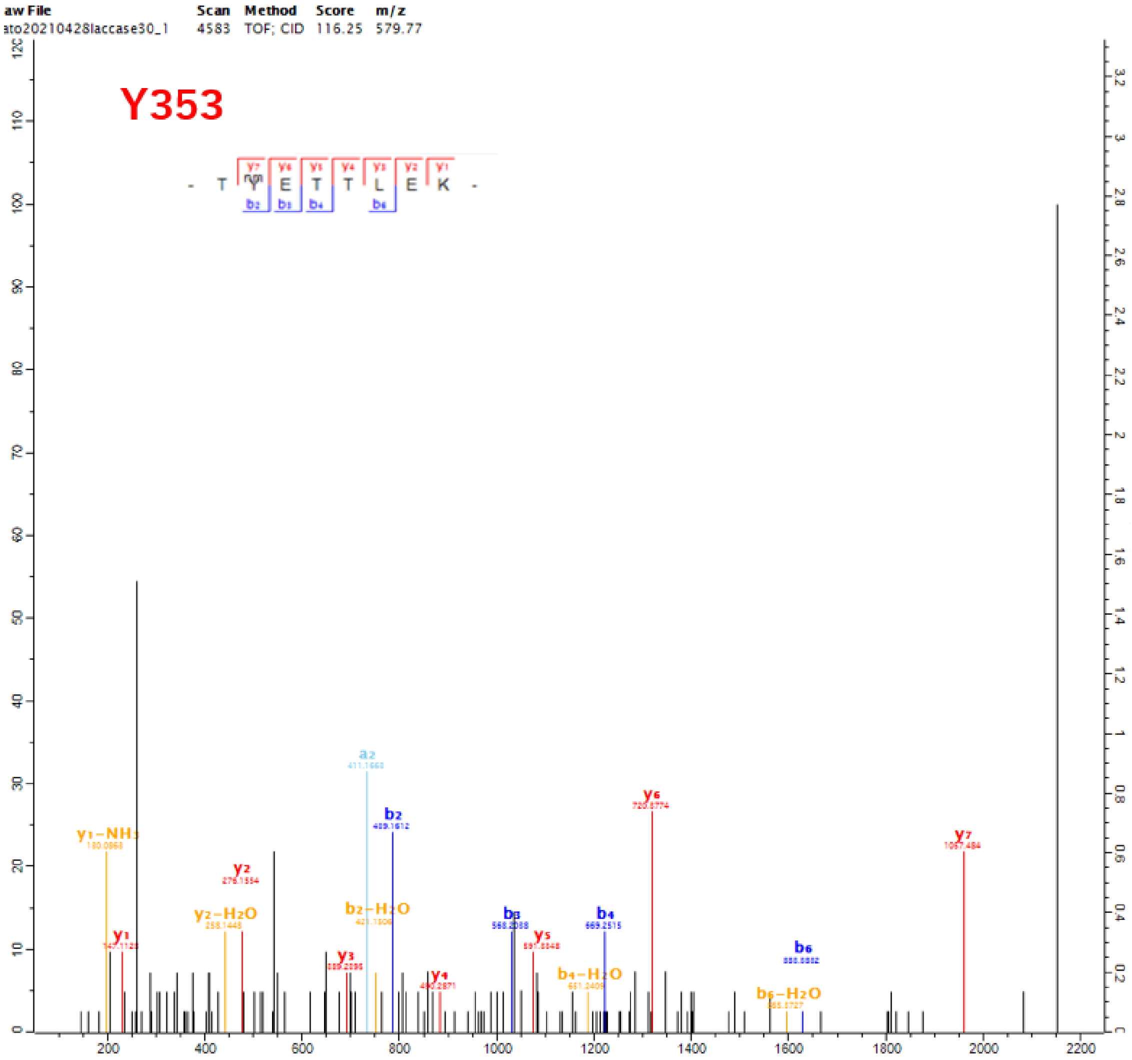

Figures S11. MS/MS analysis of the peak containing modified tyrosine shown in Figure 3J.

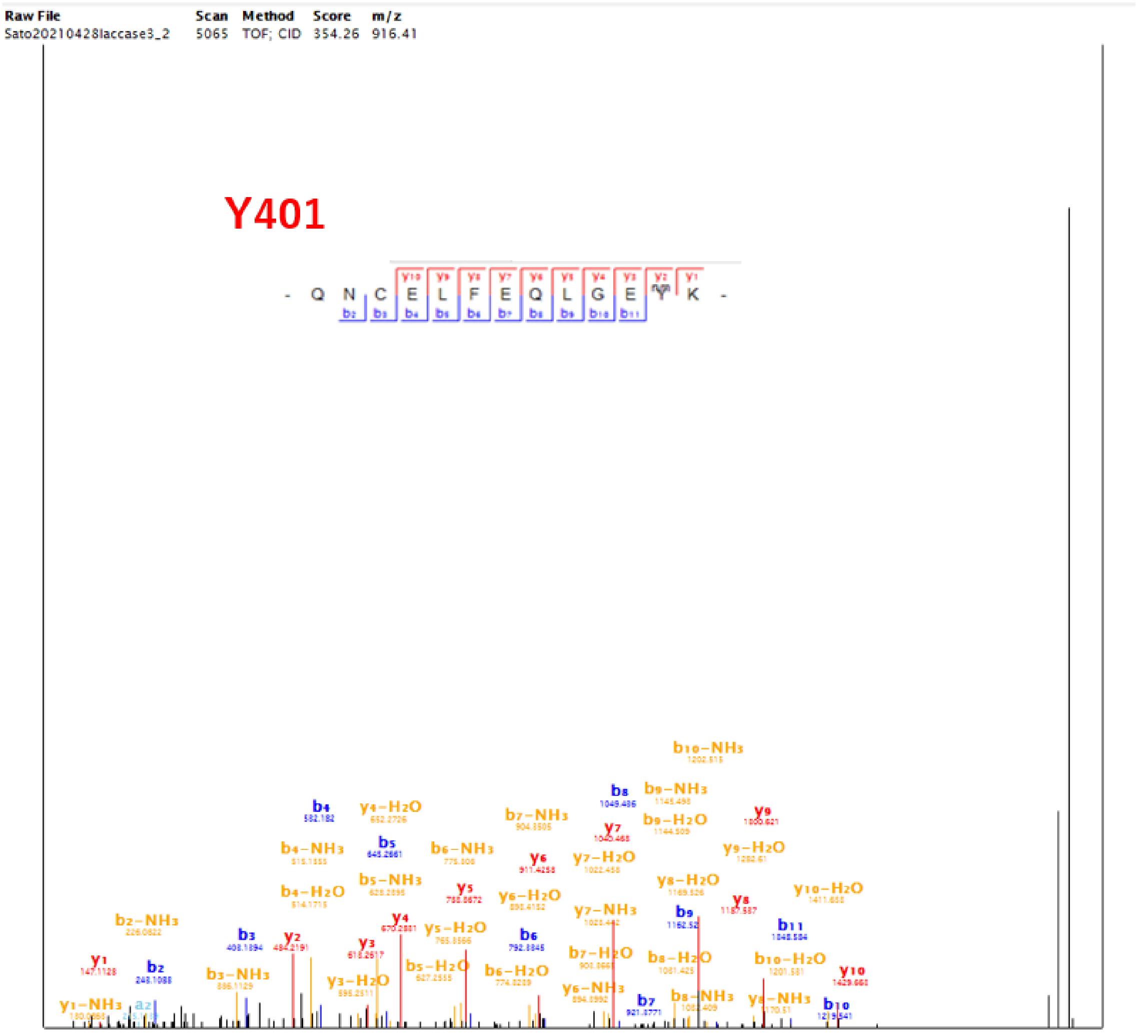

Figures S12. MS/MS analysis of the peak containing modified tyrosine shown in Figure 3K.

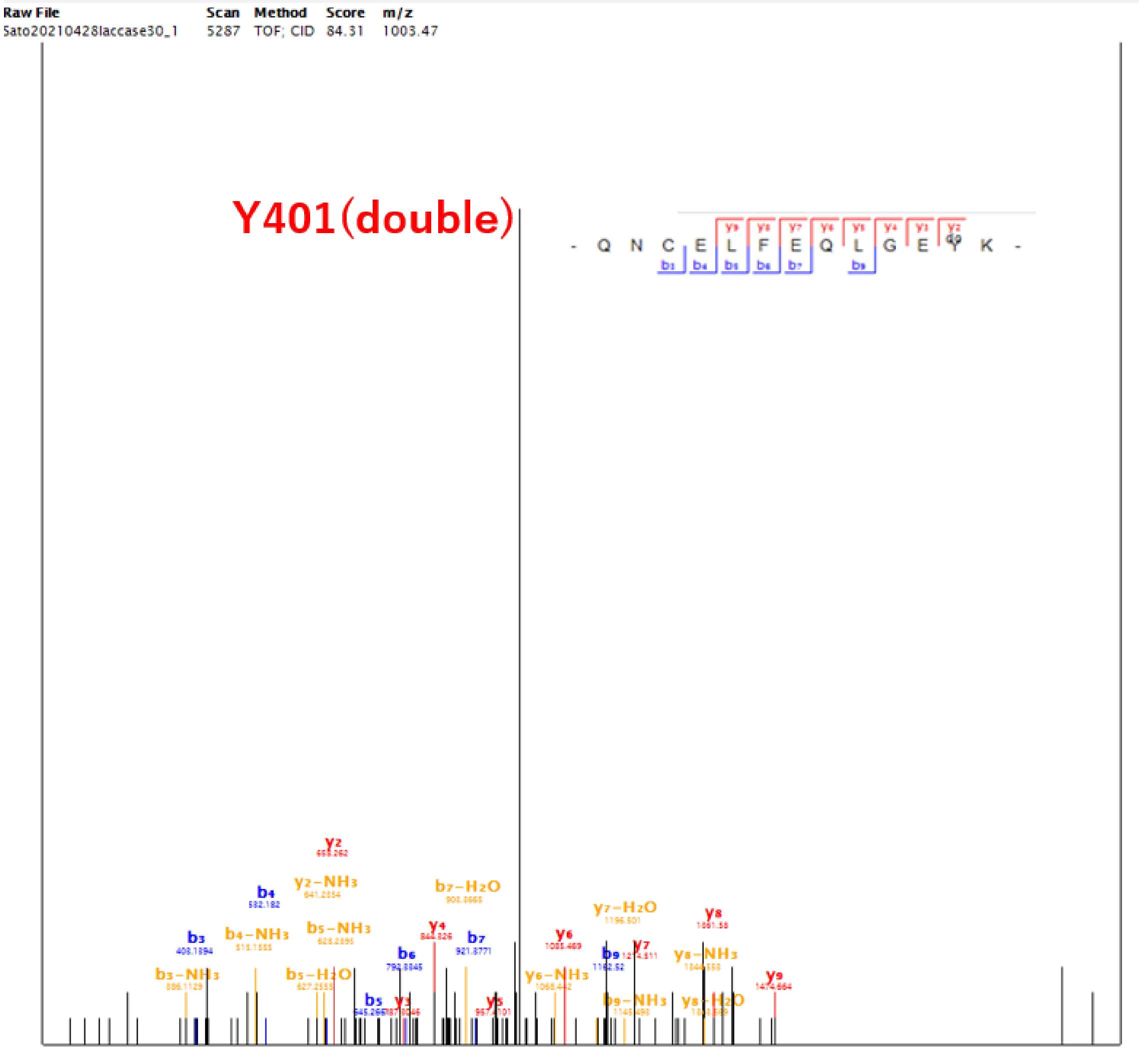

Figures S13. MS/MS analysis of the peak containing modified tyrosine shown in Figure 3L.

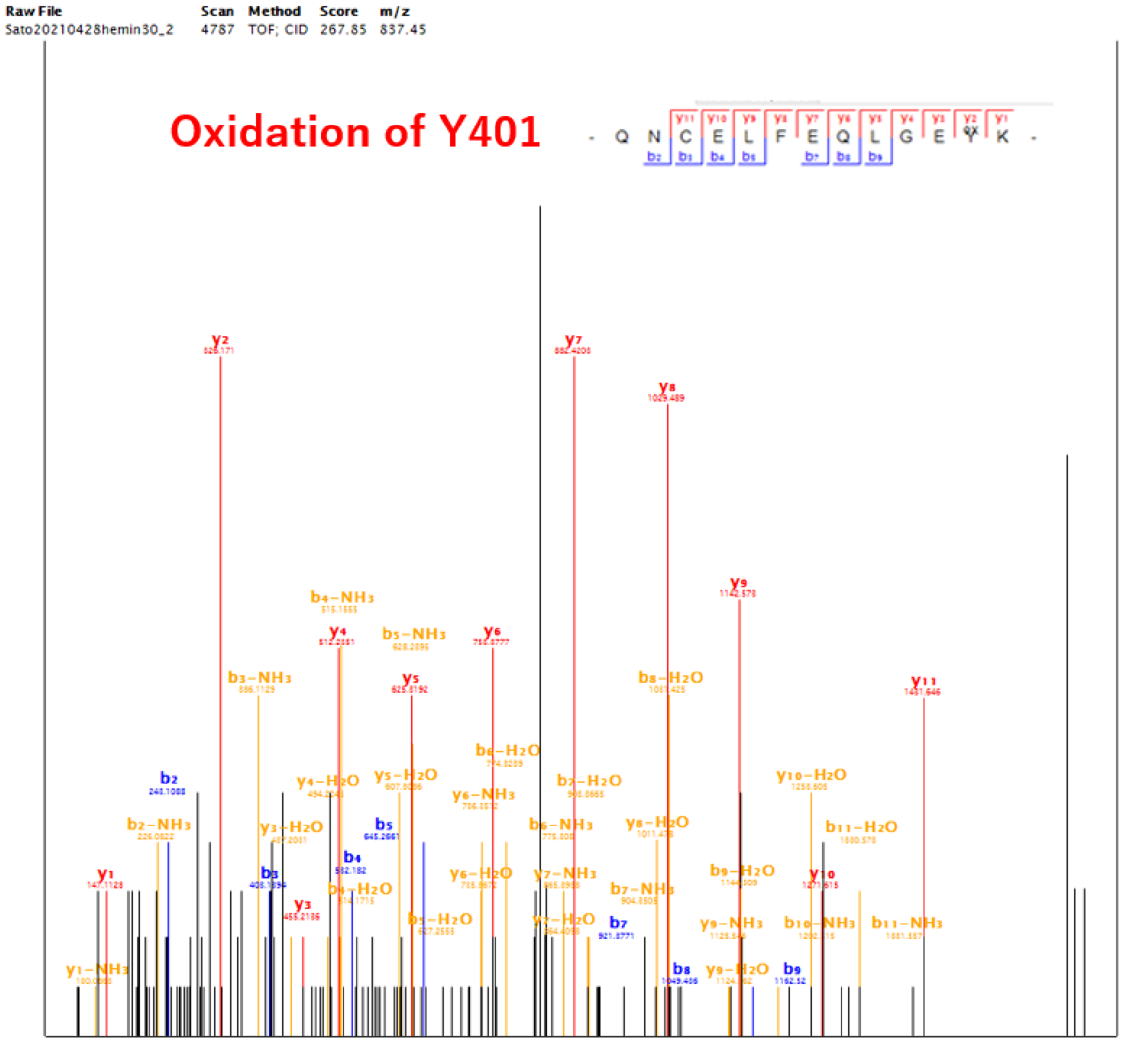

Figures S14. MS/MS analysis of the peak containing oxidized tyrosine shown in Figure 4.

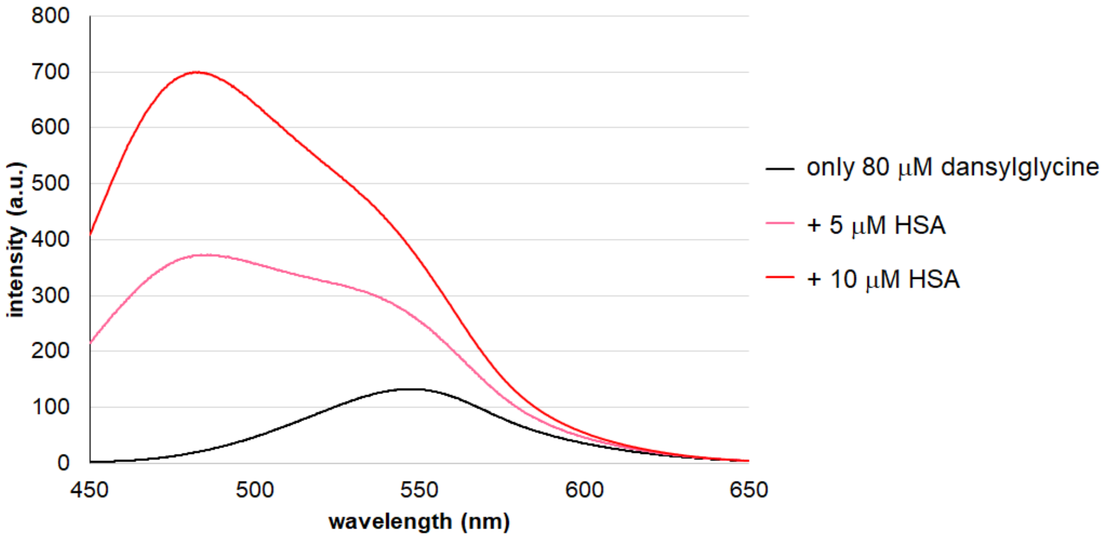

Figures S15. Change in fluorescence spectrum of dansylglycine by binding with HSA. Ex: 360 nm. Fluorescent spectrum was measured with FP-6500 (JASCO). 80 µM dansylglycine (from 100 mM DMSO stock), 5 or 10 µM HSA in 100 mM PBS (pH7.4), 25°C.

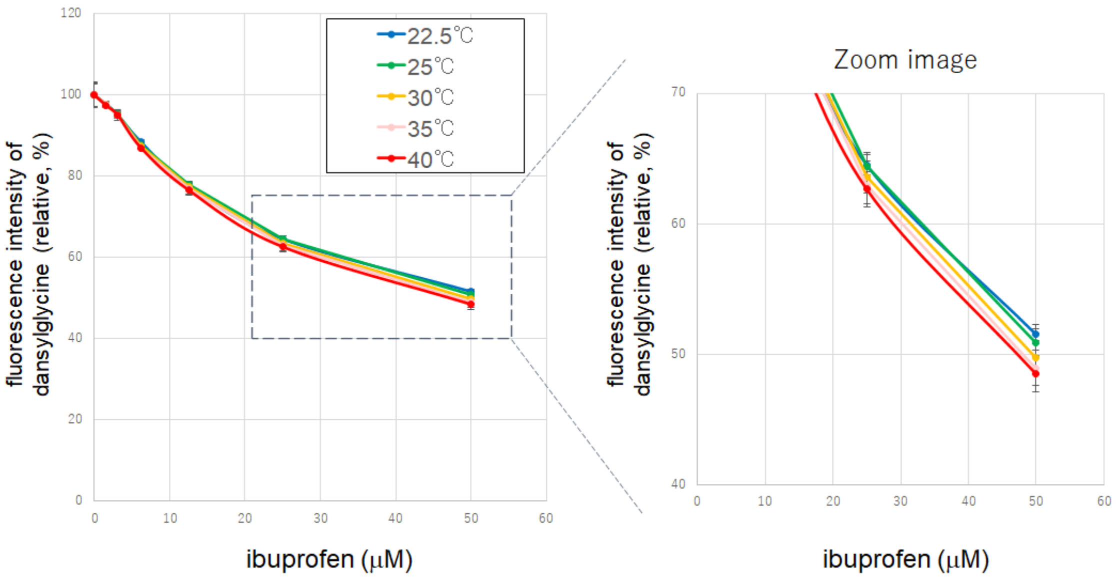

Figures S16. Temperature-dependency of the binding competition between ibuprofen and dansylglycine, Fluorescence changes due to competition between ibuprofen and dansylglycine. Error bars, mean ± SD from three independent experiments.
